# Scanxiety and quality of life around follow-up imaging in patients with unruptured intracranial aneurysms: a prospective cohort study

**DOI:** 10.1007/s00330-024-10602-0

**Published:** 2024-02-05

**Authors:** Maarten J. Kamphuis, Laura T. van der Kamp, Ruben P. A. van Eijk, Gabriel J. E. Rinkel, Johanna M. A. Visser-Meily, Irene C. van der Schaaf, Mervyn D. I. Vergouwen

**Affiliations:** 1 Department of Radiology, University Medical Center Utrecht, Utrecht University, Utrecht, The Netherlands; 2 Department of Neurology and Neurosurgery, UMC Utrecht Brain Center, University Medical Center Utrecht, Utrecht University, Utrecht, The Netherlands; 3 Biostatistics & Research Support, Julius Center for Health Sciences and Primary Care, University Medical Center Utrecht, Utrecht, The Netherlands; 4 Department of Rehabilitation, Physical Therapy Science and Sports, UMC Utrecht Brain Center, University Medical Center Utrecht, Utrecht University, Utrecht, The Netherlands

**Keywords:** Anxiety, Computed tomography angiography, Intracranial aneurysm, Magnetic resonance angiography, Quality of life

## Abstract

**Objectives:**

Patients with an unruptured intracranial aneurysm (UIA) may experience scanxiety around follow-up imaging. We studied the prevalence and temporal pattern of scanxiety, and compared quality of life (QoL) outcomes in patients with and without scanxiety.

**Methods:**

We performed a prospective cohort study in a tertiary referral center in the Netherlands between October 2021 and November 2022. We sent questionnaires to patients ≥ 18 years old undergoing UIA follow-up imaging 4 weeks before (T1), immediately after (T2), and 6 weeks after the scan (T3) to assess health-related QoL (HRQoL) and emotional functioning. At T3, we also assessed scanxiety with a purpose-designed questionnaire. We compared differences in QoL outcomes between respondents with and without scanxiety using mixed models.

**Results:**

Of 158 eligible patients, 106 (67%) participated (mean age 61 years ± 11 [standard deviation], 84 women). Sixty of the 91 respondents (66%) who completed the purpose-designed questionnaire experienced scanxiety. Of the 49 respondents who experienced scanxiety after the scan, it resolved in 22 (45%) within a day after receiving the radiology report. HRQoL did not differ between respondents with or without scanxiety. Emotional functioning was worse for respondents with scanxiety (mean Hospital Anxiety and Depression Scale sum score difference at T1, 3.6 [95% CI, 0.9–6.3]; T2, 4.1 [95% CI, 1.5–6.8]; and T3, 4.0 [95% CI, 1.5–6.5]).

**Conclusions:**

Two-thirds of the respondents experienced scanxiety around follow-up imaging, which often resolved within a day after receiving results. Patients with scanxiety had similar HRQoL but worse emotional functioning compared to patients without scanxiety. The time between the scan and receiving the results should be minimized to decrease the duration of scanxiety.

**Clinical relevance statement:**

We showed that scanxiety is common in UIA patients, and negatively associated with emotional functioning. Since scanxiety often disappears immediately after receiving the radiology report, it should be communicated to the patient as early as possible to alleviate patients’ distress.

**Key Points:**

*• Many patients with an unruptured intracranial aneurysm experience emotional distress around follow-up imaging, termed “scanxiety.”*

*• Patients with scanxiety had worse emotional functioning compared to patients without scanxiety.*

*• Scanxiety often resolved within a day after receiving the radiology report.*

**Supplementary Information:**

The online version contains supplementary material available at 10.1007/s00330-024-10602-0.

## Introduction

Patients with an unruptured intracranial aneurysm (UIA) are often followed with imaging to detect aneurysm growth [1], because preventive aneurysm treatment needs to be reconsidered after detection of aneurysm growth [2, 3]. Patients who undergo follow-up imaging may experience a temporary scan-related increase in stress and anxiety, which has been termed “scanxiety” [4]. Previous studies on scanxiety were mostly performed in patients with cancer, and found that the majority of patients experienced scanxiety, which can affect their health-related quality of life (HRQoL) [5–11]. It remains unclear whether scanxiety also plays a role in patients undergoing follow-up imaging for an untreated UIA, and if so, how long it lasts and when it is worst. Therefore, we studied the prevalence, temporal pattern, and predictors of scanxiety, and compared HRQoL and emotional functioning in patients with and without scanxiety.

## Materials and methods

### Study design and participants

Formal ethical assessment was waived by the institutional review board of University Medical Center (UMC) Utrecht, the Netherlands (METC Utrecht, 21-591/C), since surveys were a minimal burden for participants. Informed consent was obtained from all participants. This prospective study was conducted between October 2021 and November 2022 at UMC Utrecht, a tertiary referral center for patients with unruptured and ruptured intracranial aneurysms. All patients 18 years or older who were scheduled for follow-up imaging for 1 or more untreated intradural UIAs were eligible for inclusion. Patients were also included if they had 1 or more treated aneurysms (ruptured or unruptured) in addition to their untreated aneurysm. Patients were excluded if they had severe cognitive deficits or a language barrier. Six weeks before the follow-up scan, eligible patients received a letter and an information leaflet explaining the study, after which they were contacted by telephone to ask for participation. The word “anxiety” was avoided in the information leaflet to minimize bias. Questionnaires were administered at 3 time points: 4 weeks before the scan (T1), immediately after the scan and before receiving the results (T2), and 6 weeks after the scan when all patients had received the results (T3). On T1 and T3, questionnaires were sent by e-mail or post, depending on the participant’s preference. On T2, participants were approached by one of the authors (M.J.K.) immediately after the scan was completed, and the questionnaires were administered on paper. All questionnaires were written in Dutch.

### Data collection

#### Patient and aneurysm characteristics

We recorded the following patient and aneurysm characteristics: sex, age at enrollment, history of subarachnoid hemorrhage (SAH), family history of SAH, history of treatment of another intracranial aneurysm, years of follow-up at enrollment, reason for diagnosis, aneurysm size, aneurysm location, and PHASES score [12]. The PHASES score estimates the 5-year rupture risk of UIAs based on the following patient and aneurysm characteristics: population, hypertension, age, size of aneurysm, earlier SAH from another aneurysm, and site of aneurysm. The score ranges from 0 to 22, with 5-year rupture risks ranging from 0.4 to 17.8% [12]. We recorded the reason for diagnosis as “incidental” or “screening.” “Incidental” included all patients in whom the UIA was found incidentally, including the diagnosis of a UIA at the time of rupture of another aneurysm. “Screening” included patients in whom the UIA was diagnosed because of a positive family history for SAH, a patient history of autosomal polycystic kidney disease, or pre-medical screening at a commercial center. We distinguished incidentally detected UIAs from UIAs detected at screening, as we hypothesized that patients undergoing screening would be more anxious than those in whom the aneurysm was found incidentally.

We recorded the following imaging-related characteristics: imaging modality, radiology report, the number of days between performance of the scan and receiving the radiology report, management plan after follow-up imaging (preventive treatment of a UIA, retreatment of another aneurysm, continued follow-up, or end of follow-up), and time to next scheduled follow-up imaging. We recorded the radiology report as “unfavorable” or “favorable.” Unfavorable was defined as an increase in UIA size of at least 1.0 mm, a new UIA, or recanalization of a previously treated aneurysm. A radiology report was classified as favorable if the aneurysm remained stable.

#### Scanxiety

There is no uniform definition of scanxiety or validated tool to quantify it [13]. We defined scanxiety as the presence of self-reported anxiety in the period around the follow-up scan. The presence and severity of scanxiety was based on item 2 of the purpose-designed questionnaire administered at T3: “How much anxiety did you experience in the period around the scan?” (Supplementary Data 1). We measured the temporal pattern of scanxiety using this purpose-designed questionnaire, which consisted of 9 items: 4 items related to the severity of anxiety at different time points around the scan, 4 to the timing of anxiety, and 1 to respondents’ preferred imaging interval. Items relating to the severity of anxiety had 4 response categories ranging from “none” to “very much.” Two items were open-ended questions, asking respondents to indicate how much time before the scan the anxiety began to increase (item 4) and how much time after the scan the anxiety resolved (item 7). We built dependencies into the questionnaire, meaning that items were presented depending on responses to previous items (Supplementary Fig. 1).

#### Quality of life outcomes

At T1–T3, we assessed quality of life (QoL) outcomes, consisting of HRQoL and emotional functioning. We assessed HRQoL with the EuroQol 5-dimensions (EQ-5D) [14]. This questionnaire contains 5 items on the following dimensions: mobility, self-care, usual activities, pain and discomfort, and anxiety and depression. For each item, patients indicate on a 5-level scale how much difficulty they have with the activity. Responses can be converted into a single country-specific health value. The EQ-5D also contains 1 item in which patients rate their general health on the EuroQoL visual analog scale (EQ-VAS) ranging from 0 to 100, with 0 representing the worst and 100 indicating the best possible health.

We measured emotional functioning using the Hospital Anxiety and Depression Scale (HADS) [15]. This questionnaire contains 14 items with 4-level response options each, of which 7 items relate to anxiety, and 7 to depression. The questionnaire asks how patients felt during the past week. Sum scores range from 0 to 42, with 0 indicating no anxiety or depression, and 42 indicating maximum anxiety and depression.

#### Coping style

As previous studies have shown that passive coping style, a psychological personality trait, was negatively associated with HRQoL and emotional functioning in patients with neurovascular disease, we assessed whether passive coping style was also associated with scanxiety [16, 17]. Passive coping was measured at baseline (T1) with a subscale of the Utrecht Coping List (UCL-P) [18]. This questionnaire contains 7 items with 4 levels each. For each item, participants indicate how often they engage in passive coping strategies, ranging from “hardly ever” to “very often.” Sum scores range from 7 to 28, with 7 indicating low and 28 indicating high levels of passive coping.

### Statistical analysis

We dichotomized scanxiety into absent (“none”) or present (“some,” “much,” or “very much”) [5, 6, 10, 19] and explored the association of the following predictors with scanxiety: sex, PHASES score (in case a patient had multiple UIAs, we used the UIA with the highest PHASES score for analysis), history of SAH, years in follow-up, reason for diagnosis (incidental or screening), and passive coping style. We log-transformed UCL-P sum scores (passive coping style) and added 1 to all scores to deal with scores of 0. Predictors were entered into univariable and multivariable logistic regression models.

For EQ-5D, EQ-VAS, and HADS, we calculated mean sum scores with standard deviations (SDs) at each time point. Using mixed models for repeated measures, we assessed changes in EQ-5D and HADS sum scores over time for respondents with scanxiety, and compared sum scores to those without scanxiety at each time point using an interaction term between time point and scanxiety. Models had a random slope and random intercept; time was treated as a categorical variable. Results were presented as mean differences with 95% confidence intervals (CIs).

## Results

### Participants

We approached 158 eligible patients for participation, of whom 106 (67%) agreed to participate (Supplementary Fig. 2). Seven patients declined participation because they reported feelings of scan-associated distress. All participants returned at least 1 questionnaire, and 83 (78%) returned all 3 questionnaires. Sex, age, and PHASES score did not differ between participants who returned the purpose-designed questionnaire at T3 and those who did not.

### Patient and aneurysm characteristics

Table 1 shows patient and aneurysm characteristics at baseline. Most patients (84/106 [79%]) were female, and 35/106 (33%) had been treated previously for another intracranial aneurysm. At the time of follow-up imaging, patients had been in follow-up for a median of 3.2 years (IQR 1.9–5.8) for the UIA. Ninety-two patients (87%) had a UIA that was detected incidentally; 14 (13%) patients had a UIA detected by screening. In total, patients had 183 intradural aneurysms, of which 133 were untreated and unruptured, 26 were unruptured and treated, and 24 were ruptured and treated. The median PHASES score of the untreated UIAs was 4 (IQR 3–5), corresponding to a 5-year rupture risk of 0.9% [12].

**Table 1:** Baseline patient and aneurysm characteristics

Patient characteristics	*n* = 106
Female sex, *n* (%)	84 (79%)
Mean age (SD)	61 (11)
History of SAH, *n* (%)	27 (26%)
Family history of SAH, *n* (%)	13 (12%)
History of treatment of another intracranial aneurysm, *n* (%)	35 (33%)
Median number of years in follow-up (IQR)	3.2 (2.0–5.6)
Reason for diagnosis, *n* (%)	
Incidental	92 (87%)
Screening	14 (13%)
Imaging modality, *n* (%)^a^	
TOF-MRA	64 (61%)
CTA	41 (39%)
Aneurysm characteristics	*n* = 133^b^
Median size in mm (IQR)	3.5 (2.2–5.0)
Location, *n* (%)	
Anterior cerebral or communicating artery	30 (23%)
Internal carotid artery or posterior communicating artery	34 (26%)
Middle cerebral artery	55 (41%)
Posterior circulation	14 (11%)
Median PHASES score (IQR)	4 (3–5)

*IQR*, interquartile range; *SD*, standard deviation; *SAH*, subarachnoid hemorrhage; *TOF-MRA*, time-of-flight MRA; *PHASES*, risk score assessing the 5-year rupture risk of unruptured intracranial aneurysms

^a^One patient declined further imaging follow-up after enrollment

^b^Number of unruptured untreated intracranial aneurysms

### Follow-up radiology reports

Of 106 patients, 1 declined follow-up after enrollment; this patient only completed the questionnaires on T1. The other 105 underwent imaging. Thirty-six patients (34%) received a preliminary radiology report on the day of the scan from the attending neurologist or neurosurgeon. Patients received the final radiology report after a median duration of 8 days (IQR 7–14 days) after the scan. Nine patients (9%) had an unfavorable radiology report for 9 aneurysms: in 8 patients the UIA showed growth of at least 1.0 mm, and in 1 patient a previously treated aneurysm had recanalized, while the untreated UIA in this patient had remained stable. In none of the patients, the follow-up scan revealed a newly developed UIA. Of the 9 patients with an unfavorable radiology report, 8 were scheduled for aneurysm treatment. One patient was not treated, since the rupture risk of the UIA did not outweigh the risk of preventive treatment because of comorbidities and advanced age. Of the 91 patients who remained in follow-up after this scan, the next follow-up imaging was scheduled in 1 year for 45 patients (49%), 2 years for 33 patients (36%), 3 years for 7 patients (8%), and 5 years for 6 patients (7%).

### Scanxiety

**Fig. 1:**
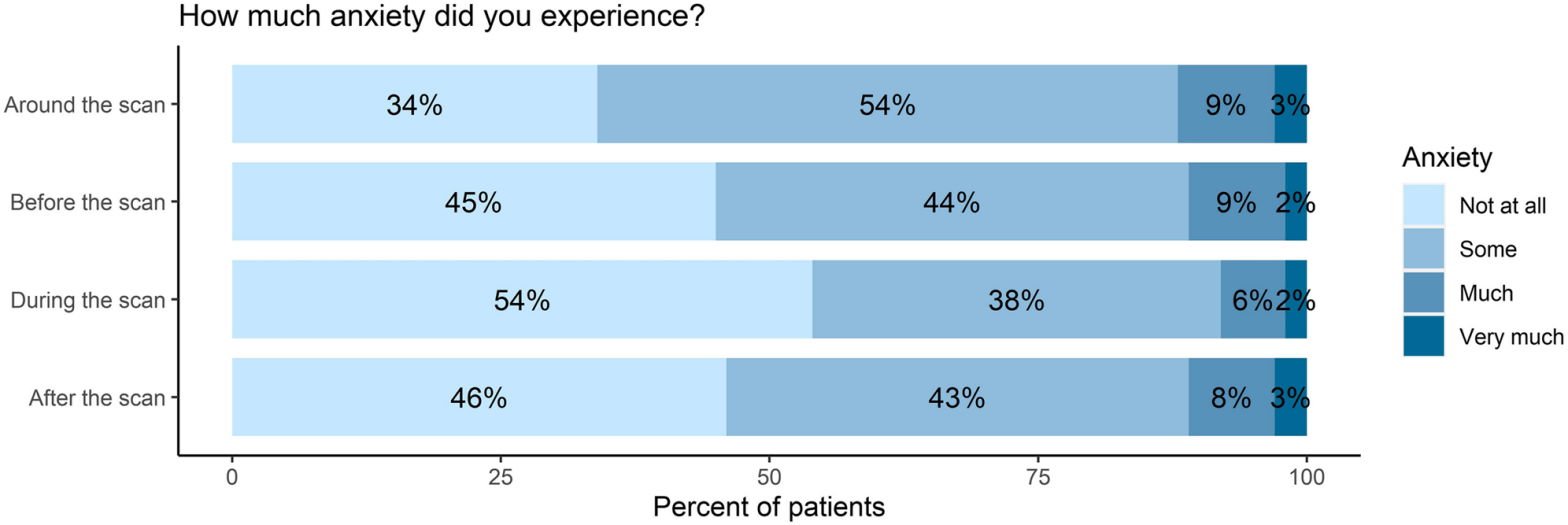
Degree of scanxiety as reported by 91 patients in the purpose-designed questionnaire at T3, 6 weeks after the follow-up scan. Bars correspond to items 1, 2, 3, and 5 of the purpose-designed questionnaire (Supplementary Data 1 and Supplementary Data 2)

Ninety-one participants returned our purpose-designed questionnaire. The number of responses differed per question because of dependencies between items (Supplementary Data 2). Sixty of 91 respondents (66%) experienced any degree of scanxiety: 49 (54%) experienced some, 8 (9%) much, and 3 (3%) very much scanxiety (Fig. 1). Fifty of 91 respondents (55%) experienced anxiety prior to the scan, 42 (46%) during the scan, and 49 (54%) after the scan.

When the 50 respondents who had scanxiety prior to the scan were asked how long before the scan the level of scanxiety started to increase, most (27/50, 54%) indicated this was 1 week before or less (Supplementary Fig. 3). Thirty-six out of 60 (60%) respondents with scanxiety experienced most anxiety between the scan and receiving the result. Of the 49 respondents who experienced scanxiety after the follow-up scan, it resolved in 32 (65%) after receiving the radiology report, and in 22 (45%) within a day after receiving the radiology report. When asked about respondents’ preferred scan interval, 42 of 91 (46%) preferred annual imaging surveillance, 19 of 91 (21%) biannual surveillance, and all others preferred longer follow-up intervals (Supplementary Fig. 4).

Patient and aneurysm characteristics stratified for the presence of scanxiety are shown in Table 2. In univariable logistic regression analysis, predictors of scanxiety were female sex (OR 3.6; 95% CI, 1.2–11.2), PHASES score (OR 0.8; 95% CI, 0.6–1.0), and passive coping style (OR 1.8; 95% CI, 1.0–3.4) (Table 2). Independent predictors of scanxiety were female sex (OR 5.7; 95% CI, 1.5–23.7) and PHASES score (OR 0.8; 95% CI, 0.6–1.0).

**Table 2:** Logistic regression analysis for predictors of scanxiety

	With scanxiety (*n* = 60)	Without scanxiety (*n* = 31)	Univariable OR (95% CI)	Multivariable OR (95% CI)
Female sex, *n* (%)	53 (88%)	21 (68%)	3.6 (1.2–11.2)	5.7 (1.5–23.7)
Median PHASES score (IQR)	4 (3–5)	5 (4–6)	0.8 (0.6–1.0)^a^	0.8 (0.6–1.0)^a^
History of SAH, *n* (%)	17 (28%)	9 (29%)	1.0 (0.4–2.6)	0.8 (0.3–2.7)
Median number of years in follow-up (IQR)	3.3 (1.9–4.9)	3.4 (1.9–6.2)	1.0 (0.9–1.1)^b^	0.9 (0.8–1.0)^b^
Diagnosed by screening, *n* (%)	10 (17%)	3 (10%)	1.9 (0.5–8.8)	1.4 (0.3–8.2)
Median log-transformed UCL-P score (IQR)	3.0 (1.0–6.0)	2.0 (0.0–3.5)	1.8 (1.0–3.4)^c^	1.7 (0.9–3.4)^c^

*IQR*, interquartile range; *SAH*, subarachnoid hemorrhage; *PHASES*, risk score assessing the 5-year rupture risk of unruptured intracranial aneurysms

^a^Per point increase in PHASES score

^b^Per year increase in follow-up

^c^Per point increase in log-transformed UCL-P sum score

### QoL outcomes

**Fig. 2:**
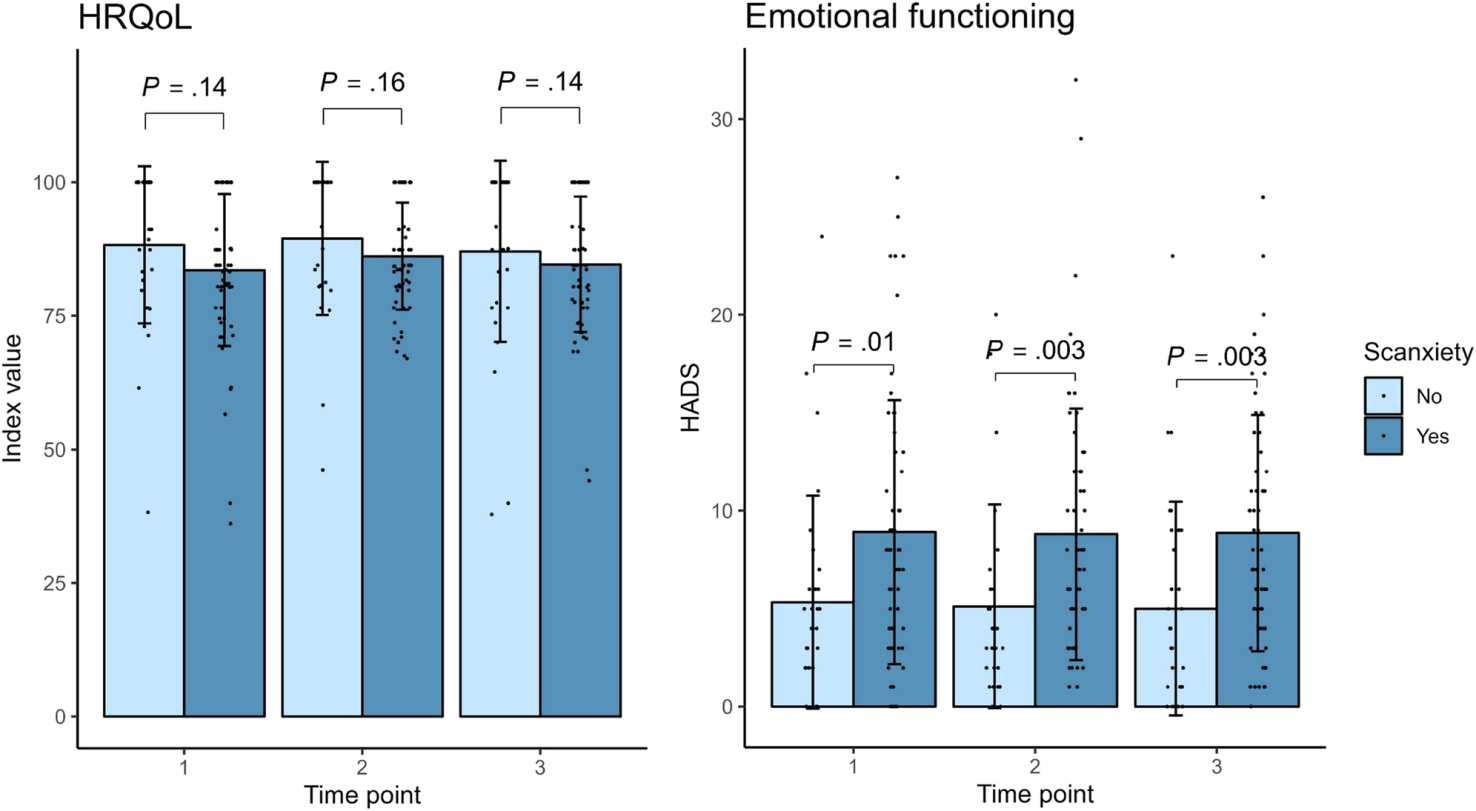
Scanxiety and quality of life outcomes. HRQoL, health-related quality of life; HADS, Hospital Anxiety and Depression Scale. Bars indicate means; error bars indicate standard deviations. *P* values represent differences between patients with and without scanxiety at every time point, obtained with mixed models for repeated measures. The presence of scanxiety is defined as any degree of scanxiety as indicated on item 2 of the purpose-designed questionnaire (Supplementary Data 1 and Supplementary Data 2)

Mean sum scores for HRQoL and HADS over time, stratified for the presence of scanxiety, are shown in Supplementary Table 1. Estimated mean HRQoL and HADS sum scores did not change over time for respondents with scanxiety (Supplementary Table 2). When comparing respondents with scanxiety and without scanxiety at each time point, HRQoL did not differ, but emotional functioning was worse for those with scanxiety (mean HADS sum score difference at T1, 3.6 [95% CI, 0.9 to 6.3]; at T2, 4.1 [95% CI, 1.5 to 6.8]; and at T3, 4.0 [95% CI, 1.5 to 6.5]) (Fig. 2).

## Discussion

Two-thirds of the respondents experienced some degree of scanxiety around follow-up imaging for a UIA; the majority experienced the highest level of scanxiety between the scan and receiving the radiology report, and it often resolved within a day after receiving the radiology report. HRQoL did not differ between respondents with and without scanxiety, but emotional functioning was lower in respondents with scanxiety at all time points.

Most previous studies on scanxiety were performed in patients with cancer [5–7, 10, 11] or in patients undergoing an imaging procedure for any diagnosis [19–21], and found a widely varying prevalence because of heterogeneity in scanxiety definitions. One study in 222 patients with advanced cancer used a definition similar to ours, and found a prevalence of 55% [6], which is only slightly lower than the prevalence we found. Studies in patients with cancer have evaluated the temporal pattern of scanxiety, and also found that scanxiety peaked in the time period between imaging and receiving the radiology report, and dropped soon after receiving the radiology report [5–7, 11].

Data on scanxiety in patients with an intracranial aneurysm are scarce. In one study, 120 patients undergoing a follow-up MRA > 4.5 years after coiling of an intracranial aneurysm were asked to complete a questionnaire on anxiety and depression within 4 weeks after the scan and 3 months after the scan [22]. One in 4 patients indicated that the scan caused them to think more about the coiled aneurysm and 1 in 10 reported that they were more afraid of recurrent SAH at the time of the scan [22]. In a study on the psychological and functional impact of having a UIA smaller than 7 mm, 3 of 33 patients in the study (9%) reported negative experiences, such as panic attacks, during the imaging procedure [23]. Eighteen percent of the patients mentioned that waiting for the radiology report was a source of anxiety [23]. These studies cannot be directly compared with ours because we used a different definition of scanxiety, but they suggest that patients with an intracranial aneurysm are prone to experience scanxiety, which is in line with our findings.

We found that female sex was an independent predictor of scanxiety, which is consistent with a previous study in patients with advanced cancer which reported a higher scanxiety prevalence in females [6]. A study in 103 patients with lung cancer [10] and a study in 70 patients with lymphoma [11] found that females tended to experience more severe scan-associated psychologic distress than males [10, 11]. Similar results were found in a study that included 488 patients undergoing an imaging procedure [21], and a study in 33 UIA patients [23]. We found that patients with a higher PHASES score, and therefore a higher risk of aneurysm rupture, were less likely to experience scanxiety. It may be that patients with a higher risk of aneurysm rupture were more involved in the shared decision to follow the aneurysm with imaging and therefore less often report feelings of scanxiety. It is also possible that the effect we found is the result of bias in the treatment decision: perhaps anxious patients tend to choose preventive treatment more often than those who are less anxious. In line with this, a previous study reported that patients scheduled for preventive occlusion of their UIA had lower emotional functioning at baseline compared to those who were followed with imaging, even before consulting with the clinician to discuss potential treatment [17].

Passive coping style was a predictor of scanxiety, but not independent of sex and PHASES score. A study in 99 UIA patients identified passive coping as an independent predictor of QoL outcomes, after correction for variables such as sex and PHASES score [17]. Thus, passive coping seems to be more strongly associated with HRQoL and general emotional functioning than with scanxiety specifically.

Respondents with scanxiety had lower levels of emotional functioning compared to respondents without scanxiety at all 3 time points, which is consistent with the results of previous studies in patients with cancer [5, 6]. Since we did not find a change in emotional functioning over time in participants with scanxiety, this suggests that the lower level of emotional functioning was not caused by scanxiety, but rather that respondents with poorer emotional functioning are more prone to experience scanxiety. Respondents with and without scanxiety had similar levels of HRQoL at all time points; HRQoL therefore seems to reflect general well-being rather than scanxiety specifically, and only 1 of 5 items in the EQ-5D targets anxiety and depression [14].

We measured general anxiety and depression using the HADS [15], which is a validated tool to quantify anxiety and depression [15, 24] and has been used in previous studies on scanxiety [6, 13, 23]. An alternative measurement tool is the state-trait anxiety inventory, which measures temporary anxiety states and long-lasting traits separately [25]. Since the HADS does not make this distinction, it may be less sensitive to temporary states. However, its score has been shown to vary over time within patients in longitudinal studies [17, 26], and it is therefore likely that we would have observed a time-dependent effect of scanxiety on emotional functioning if there was one.

The main strength of this study is its prospective longitudinal design and large sample size compared to previous studies [5, 10, 11, 23], and the use of standardized questionnaires, which contributes to its generalizability. We also need to address a few limitations. First, selection bias may have played a role: 7 patients did not participate in this study because they indicated feelings of scan-associated distress. We do not believe that this would have changed the conclusions of the study, given the relatively small number of patients who declined participation for this reason. Second, we assessed scanxiety with our purpose-designed questionnaire at a single time point 6 weeks after the scan, which may have introduced recall bias. Third, not all participants completed questionnaires at T3, including the purpose-designed questionnaire. This could have introduced bias, for example if participants experiencing high levels of scanxiety did not complete questionnaires at T3. Fourth, this is a monocenter study which limits its generalizability. Management decisions for UIAs vary between centers and countries [27, 28], leading to a different selection of patients with an untreated UIA, which may affect their QoL outcomes [17].

In conclusion, two-thirds of the respondents experienced scanxiety, which was worst between the scan and receiving results, and often resolved within a day after receiving results. Patients with scanxiety had lower levels of emotional functioning. The results of this study imply that the time between the scan and receiving the result should be minimized to decrease the duration of scanxiety.

## Supplementary Information

Below is the link to the electronic supplementary material.

Supplementary file1 (DOCX 552 KB)

## Abbreviations

EQ-5D: EuroQol 5-dimensions
HADS: Hospital Anxiety and Depression Scale
HRQoL: Health-related quality of life
PHASES: Risk score to assess the 5-year rupture risk of unruptured intracranial aneurysms
QoL: Quality of life
SAH: Subarachnoid hemorrhage
TOF-MRA: Time-of-flight MRA
UCL-P: Utrecht Coping List–passive scale
UIA: Unruptured intracranial aneurysm
VAS: Visual analog scale

## Declarations

### Guarantor

The scientific guarantor of this publication is Mervyn D.I. Vergouwen.

### Statistics and biometry

Ruben P.A. van Eijk has significant statistical expertise and kindly provided statistical advice for this manuscript.

### Study subjects or cohorts overlap

No study subjects or cohorts have been previously reported.

### Methodology

• prospective

• observational

• performed at one institution
