# Supplementary material for: Scanxiety and quality of life around follow-up imaging in patients with unruptured intracranial aneurysms: a prospective cohort study
Source: Eur Radiol. 2024 Feb 5;34(9):6018–25. doi: 10.1007/s00330-024-10602-0 (PMC11364567; doi:10.1007/s00330-024-10602-0)
Supplement: Supplementary file 1 — Supplementary file1 (DOCX 552 KB) [file 330_2024_10602_MOESM1_ESM.docx]

**SUPPLEMENTARY MATERIAL**

**Scanxiety and quality of life around follow-up imaging in patients with unruptured intracranial aneurysms: a prospective cohort study**

*Table of Contents*

**Supplementary Data 1.** Purpose-designed questionnaire.

**Supplementary Figure 1.** Flow through the purpose-designed questionnaire depending on responses to previous items.

**Supplementary Figure 2.** Flowchart of eligible patients.

**Supplementary Data 2.** Responses to purpose-designed questionnaire.

**Supplementary Figure 3.** Timing of scanxiety as reported in the purpose-designed questionnaire.

**Supplementary Figure 4.** Respondents’ preferred scan interval as reported in the purpose-designed questionnaire (n= 91).

**Supplementary Table 1.** Mean QoL outcomes over time stratified for scanxiety.

**Supplementary Table 2.** Estimated mean QoL outcomes over time stratified for scanxiety.

**Supplementary Data 1.** Purpose-designed questionnaire**.**

Questions below were translated from Dutch.

The next questions are about your experiences with the most recent scan for your brain aneurysm. Please select the options that fit best.

1. How much anxiety did you experience during the scan?

☐ None
☐ Some
☐ Much
☐ Very much

2. How much anxiety did you experience in the period around the scan?
☐ None – proceed to question 9
☐ Some – proceed to question 3
☐ Much – proceed to question 3
☐ Very much – proceed to question 3

The next questions are about different moments around the scan at which you may have felt anxious due to the scan.

3. How much anxiety did you experience before the scan?

☐ None – proceed to question 5
☐ Some – proceed to question 4
☐ Much – proceed to question 4
☐ Very much – proceed to question 4

4. How much time before the most recent scan did the anxiety start to increase? Please fill in a specific number in days or weeks. E.g. ‘3 weeks’.

..................................................

5. How much anxiety did you experience after the scan?

☐ None – proceed to question 8
☐ Some – proceed to question 6
☐ Much – proceed to question 6
☐ Very much – proceed to question 6

6. At which moment had the anxiety resolved?

☐ After the scan, but before receiving the results – proceed to question 8
☐ After receiving the results – proceed to question 7
☐ It has never resolved – proceed to question 8

7. How much time after receiving scan results had the anxiety resolved? Please fill in a specific number in days or weeks. E.g. ‘3 weeks’.
..................................................

8. At which moment did you experience most anxiety?

☐ Before the scan

☐ During the scan

☐ Between the scan and receiving the results

☐ After receiving the results

You have a brain aneurysm that we monitor with scans to see if it grows. If the aneurysm grows, we can decide together with you to treat it preventively. The advantage of more scans is that changes in aneurysms size can be detected earlier. A disadvantage is that you will be reminded of the aneurysm more frequently.

9. Which interval would you prefer for follow-up scans of your brain aneurysm?

☐ Every 6 months

☐ Every year

☐ Every 2 years

☐ Every 3 years

☐ Every 5 years

☐ Other, please fill in a specific number of months or years: ……………….

**Supplementary Figure 1.** Flow through the purpose-designed questionnaire depending on responses to previous items.
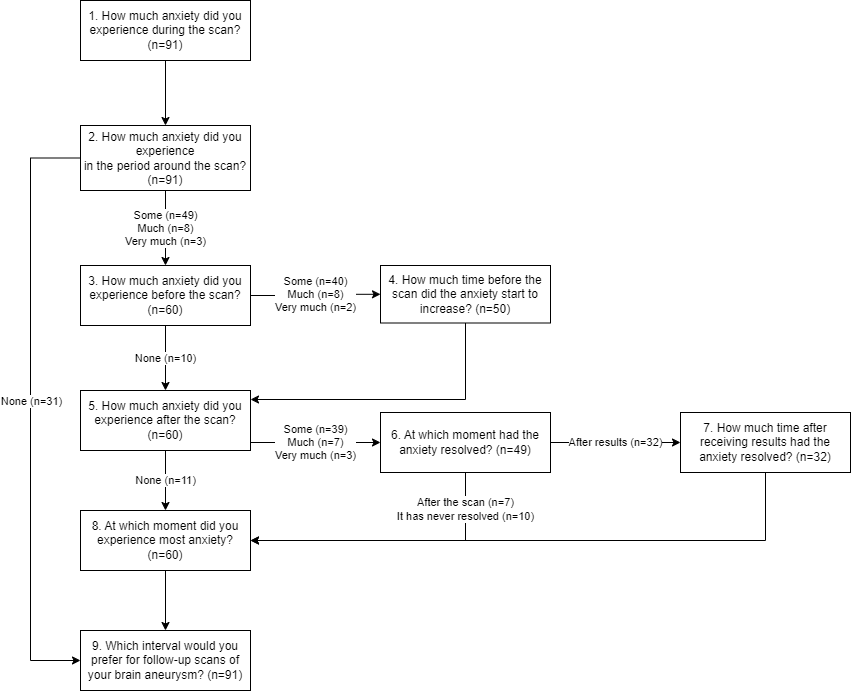


**Supplementary Figure 2.** Flowchart of eligible patients.


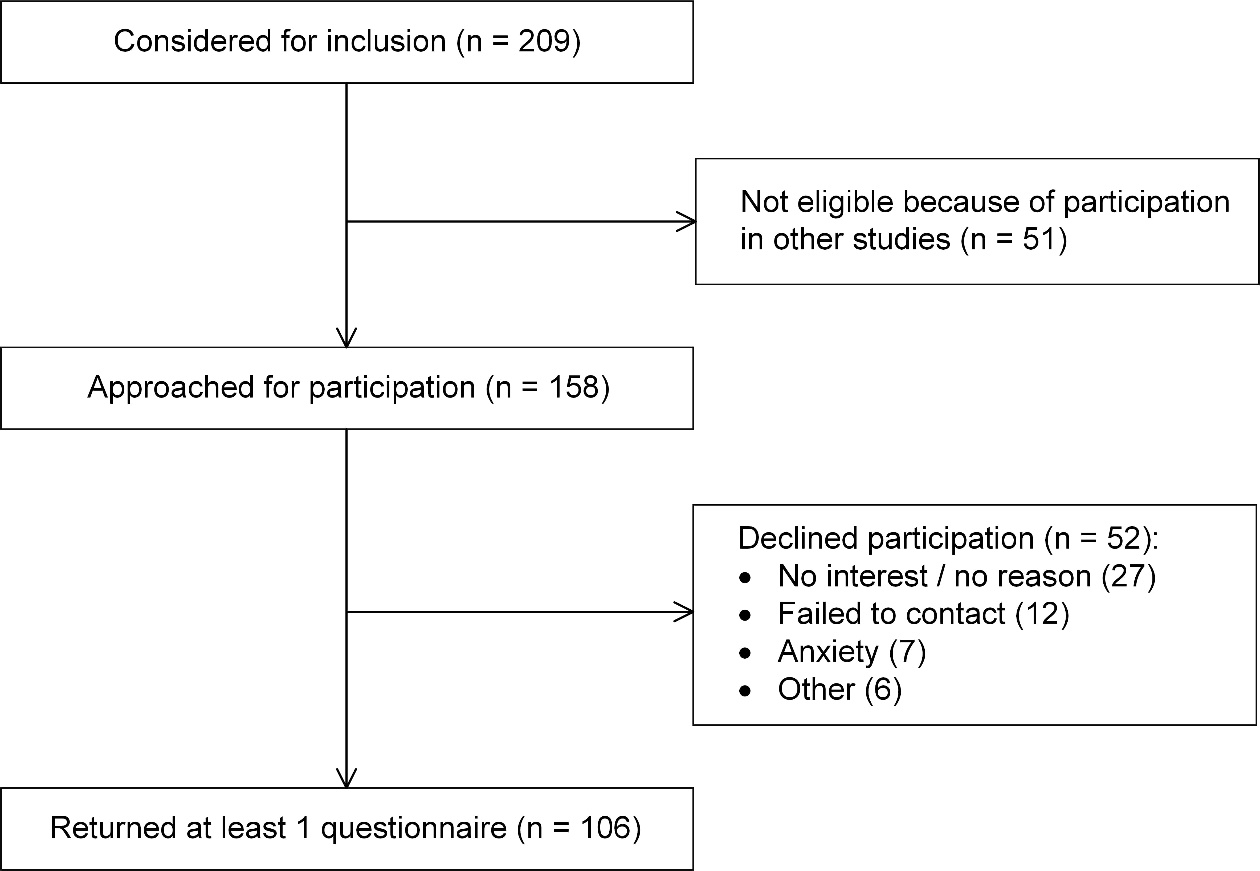


**Supplementary Data 2.** Responses to purpose-designed questionnaire.

| 1 | How much anxiety did you experience during the scan? | N=91 |
| --- | --- | --- |
|  | ☐ None | 49 |
|  | ☐ Some | 35 |
|  | ☐ Much | 5 |
|  | ☐ Very much | 2 |
| 2 | How much anxiety did you experience in the period around the scan? | N=91 |
|  | ☐ None | 31 |
|  | ☐ Some | 49 |
|  | ☐ Much | 8 |
|  | ☐ Very much | 3 |
| 3 | How much anxiety did you experience before the scan? | N=60 |
|  | ☐ None | 10 |
|  | ☐ Some | 40 |
|  | ☐ Much | 8 |
|  | ☐ Very much | 2 |
| 4 | How much time before the most recent scan did the anxiety start to increase? | N=50 |
|  | ☐ NA | 5 |
|  | ☐ >4 weeks | 4 |
|  | ☐ 2-4 weeks | 7 |
|  | ☐ 1-2 weeks | 7 |
|  | ☐ 4-7 days | 11 |
|  | ☐ 0-3 days | 16 |
| 5 | How much anxiety did you experience after the scan? | N=60 |
|  | ☐ None | 11 |
|  | ☐ Some | 39 |
|  | ☐ Much | 7 |
|  | ☐ Very much | 3 |
|  |  |  |
| 6 | At which moment had the anxiety resolved? | N=49 |
|  | ☐ After the scan, but before receiving the results | 7 |
|  | ☐ After receiving the results | 32 |
|  | ☐ It has never resolved | 10 |
| 7 | How much time after receiving scan results had the anxiety resolved? | N=49 |
|  | ☐ NA | 17 |
|  | ☐ 0-1 day | 22 |
|  | ☐ 2-7 days | 4 |
|  | ☐ 1-2 weeks | 6 |
| 8 | At which moment did you experience most anxiety? | N=60 |
|  | ☐ NA | 1 |
|  | ☐ Before the scan | 11 |
|  | ☐ During the scan | 5 |
|  | ☐ Between the scan and receiving the results | 36 |
|  | ☐ After receiving the results | 7 |
| 9 | Which interval would you prefer for follow-up scans of your brain aneurysm? | N=91 |
|  | ☐ NA | 2 |
|  | ☐ Every 6 months | 19 |
|  | ☐ Every year | 42 |
|  | ☐ Every 2 years | 20 |
|  | ☐ Every 3 years | 2 |
|  | ☐ Every 5 years | 6 |

**Supplementary Figure 3.** Timing of scanxiety as reported in the purpose-designed questionnaire.
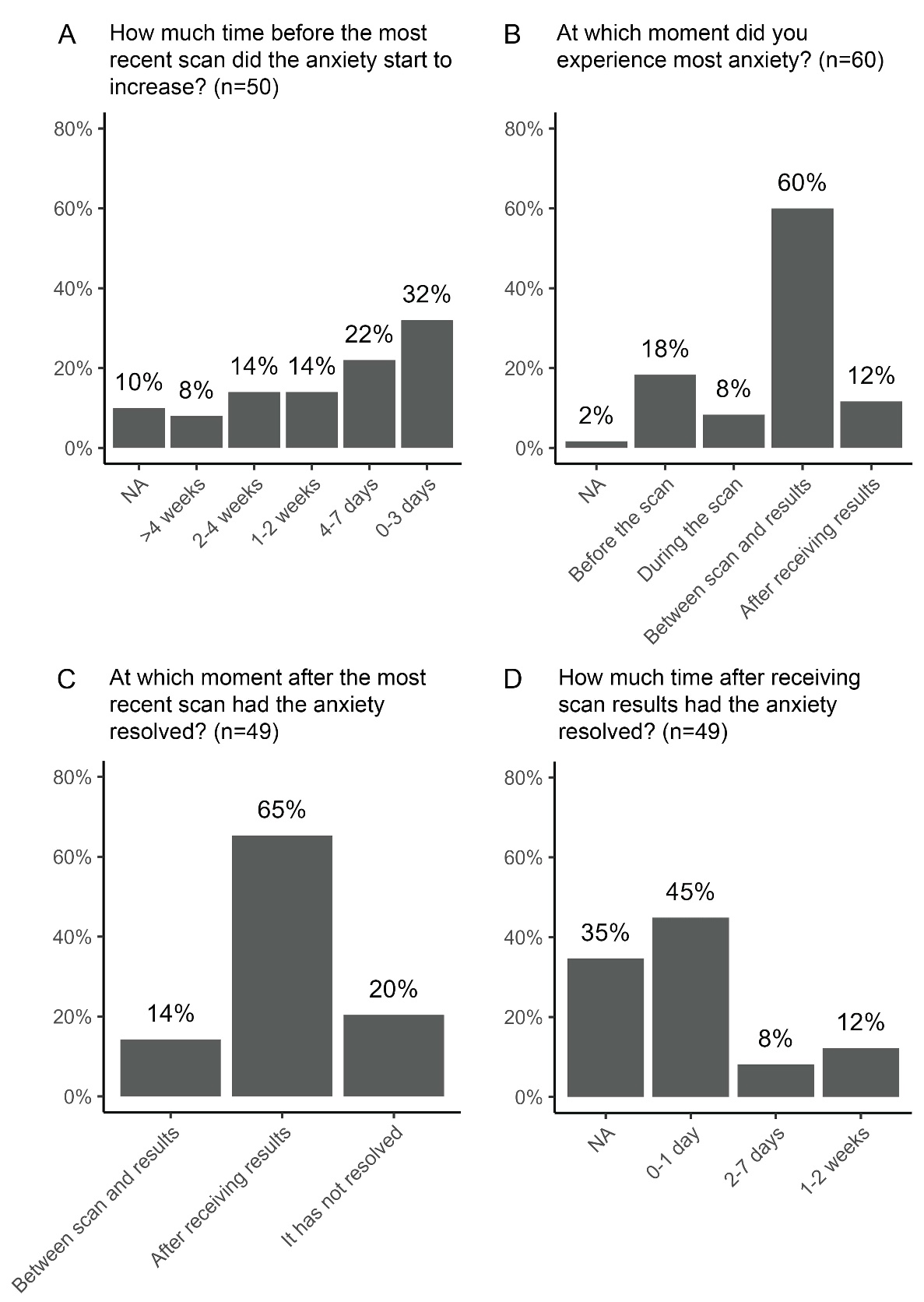


Panels A-D correspond to items 4, 8, 6 and 7 of the purpose-designed questionnaire, respectively (Online Supplemental Data). Responses are marked ‘NA’ if they could not be interpreted or if the respondent did not complete the item. Item in D was only presented to patients who responded with ‘After receiving results’ to item in C. Patients who responded with ‘between scan and results’ or ‘it has not resolved’, are represented with ‘NA’ in D.

**Supplementary Figure 4.** Respondents’ preferred scan interval as reported in the purpose-designed questionnaire (n= 91).


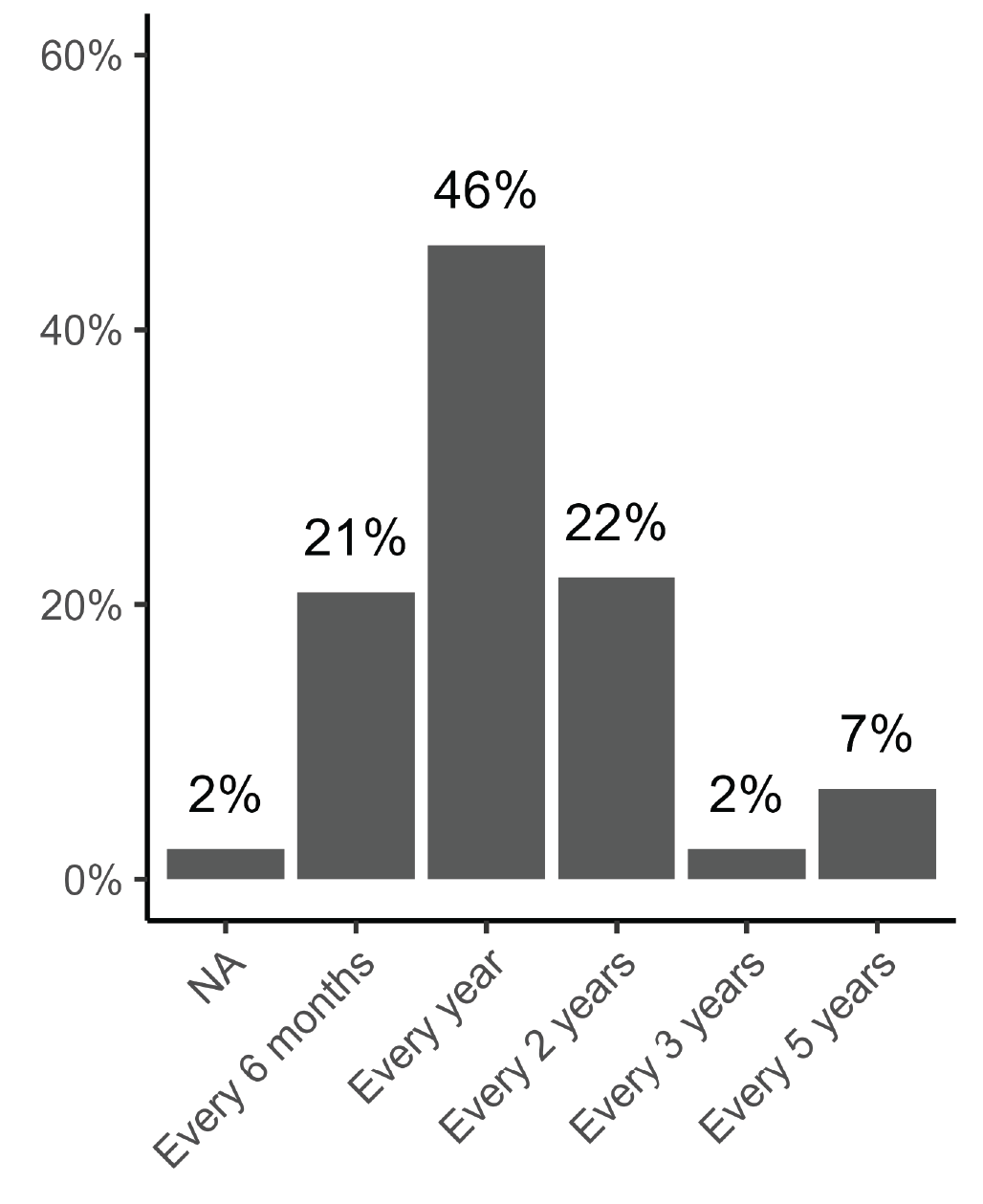


Based on item 9 of the purpose-designed questionnaire.

**Supplementary Table 1.** Mean QoL outcomes over time stratified for scanxiety.

|  | HRQoL | | | | Emotional functioning | |
| --- | --- | --- | --- | --- | --- | --- |
|  | EQ-5D | | EQ-VAS | | HADS | |
|  | n | Mean (SD) | n | Mean (SD) | n | Mean (SD) |
| Four weeks before the scan  With scanxiety  Without scanxiety | 98  57  30 | 84.2 (15.8)  83.6 (14.2)  88.3 (14.7) | 97  57  30 | 78.7 (13.4)  77.4 (13.5)  82.0 (11.4) | 97  57  30 | 7.98 (6.92)  8.91 (6.73)  5.33 (5.43) |
| Immediately after the scan  With scanxiety  Without scanxiety | 96  58  26 | 84.6 (15.8)  86.2 (10.0)  89.5 (14.3) | 96  58  26 | 78.5 (15.7)  78.6 (13.2)  84.6 (12.0) | 95  57  26 | 8.28 (6.95)  8.81 (6.41)  5.12 (5.19) |
| Six weeks after the scan  With scanxiety  Without scanxiety | 89  59  30 | 85.5 (14.2)  84.6 (12.7)  87.1 (17.0) | 89  59  30 | 79.4 (13.0)  77.0 (12.7)  84.1 (12.5) | 89  59  30 | 7.56 (6.09)  8.86 (6.02)  5.00 (5.45) |

HRQoL, health-related quality-of-life; EQ-VAS, EuroQoL visual analog scale; HADS, hospital anxiety and depression scale; SD, standard deviation. Numbers do not add up in all cases, because patients may have completed the EQ-5D or HADS, but not the purpose-designed questionnaire and vice versa.

**Supplementary Table 2.** Estimated mean QoL outcomes over time stratified for scanxiety.

|  | HRQoL (EQ-5D)  Coefficient (95% CI) | *P* value | Anxiety and depression (HADS)  Coefficient (95% CI) | *P* value |
| --- | --- | --- | --- | --- |
| Intercept | 83.8 (80.1 to 87.5) |  | 9.0 (7.4 to 10.6) |  |
| With scanxiety^a^  Four weeks before scan  Directly after scan  Six weeks after scan | Ref.  2.4 (-0.7 to 5.4)^b^  0.8 (-2.5 to 4.0) | .14  .65 | Ref.  -0.0 (-1.0 to 1.0)  -0.0 (-0.9 to 0.9) | .97  .94 |
| Without scanxiety^a,c^  Four weeks before scan  Directly after scan  Six weeks after scan | 4.8 (-1.5 to 11.1)  3.7 (-1.4 to 8.8)  2.5 (-3.7 to 8.7)^d^ | .14  .16  .44 | -3.6 (-6.3 to -0.9)  -4.1 (-6.8 to -1.5)  -4.0 (-6.5 to -1.5) | .01  .003  .003 |

HRQoL, health-related quality-of-life; EQ-5D, EuroQol 5-dimensions; HADS, hospital anxiety and depression scale; CI, confidence interval.

^a^ Scanxiety was categorized as present if the respondent indicated any degree of scanxiety on item 2 of the purpose-designed questionnaire.

^b^ Patients with scanxiety had on average 2.4% higher HRQoL directly after the scan compared to four weeks before the scan.

^c^ Compared to patients with scanxiety per timepoint.

^d^ To adjust for both timepoint and the presence of scanxiety, add the mean differences for timepoint and scanxiety to the intercept. For example, six weeks after the scan a patient without scanxiety on average had a HRQoL of 83.8 + 0.8 + 2.5 = 87.1%.
